# Predictors of childhood food allergy

**DOI:** 10.1186/2045-7022-1-S1-P24

**Published:** 2011-08-12

**Authors:** Suleiman Al-Hammadi, Taoufik Zoubeidi

**Affiliations:** 1 UAE University, Pediatrics, Al-Ain, United Arab Emirates; 2 UAE University, Statistics, Al-Ain, United Arab Emirates

## Background

Food allergy is a relatively common pediatric problem, affecting 5-8% of young children. Its occurrence is strongly associated with other atopies, and it is a main cause of anaphylaxis in children. The disease, like other atopies, appears to be familial. Genetic and environmental factors (e.g., diet in infancy, aeroallergens and geography) predispose to food allergy.

## Methods

This a cross sectional study. To assess which characteristics were associated with food allergy, we conducted simple logistic regressions of each type of atopy with food allergy as the dependent variable. A p<0.05 was considered significant. In the data modeling, we did not assume any hierarchical effects of families and schools on the food allergy status of participating children. Therefore, logistic regression with fixed effects only was used in modeling the data. Stepwise multilogistic regression with forward entry was used to determine a subset of characteristics that predicts best the presence of food allergy within the target population. Data were analyzed using SPSS statistical package (version 18).

## Results

Three hundred ninety-seven children (205 female) were enrolled on the study. The mean (SD) age was 7.2 (1.1) years. Two hundred seventy-one (68.2%) were fed solely human milk, 122 (30.7%) were fed human milk supplemented with cow milk protein-based formulas. Significant associations were present between childhood food allergy and personal atopic dermatitis (p<0.001), asthma (p <0.001) and rhino-conjunctivitis (p<0.05). Significant associations present between childhood food allergy and an immediate family member with food allergy, asthma, atopic dermatitis, or rhino-conjunctivitis. The best predictors of food allergy were personal atopic dermatitis (p=0.000), personal asthma (p=0.000), father with atopic dermatitis (p=0.005) and father with rhino-conjunctivitis (p=0.012).

## Conclusions

The data confirm childhood food allergy is significantly associated with personal or family atopy (asthma, eczema or rhino-conjunctivitis). Similarly, paternal atopic dermatitis and rhino-conjunctivitis are shown here to be among the best indicators of childhood food allergy.

